# Barriers and drivers of psychosocial risk assessments in German micro and small-sized enterprises: a qualitative study with owners and managers

**DOI:** 10.1186/s12889-021-11416-1

**Published:** 2021-07-12

**Authors:** Valeria Pavlista, Peter Angerer, Mathias Diebig

**Affiliations:** Heinrich-Heine University, Institute for Occupational, Social and Environmental Medicine, Centre for Health and Society; Medical Faculty, 40225 Dusseldorf, Germany

**Keywords:** Psychosocial stress, Working conditions, Job stress, Health promotion, Risk assessment, Small firms, Micro- and small enterprises, Qualitative research, Occupational health and safety, Health promotion implementation

## Abstract

**Background:**

The negative effect of unfavorable working conditions and long-term work stress on health has been demonstrated in previous research. To address these challenges, systematic approaches such as psychosocial risk assessments (PRA) have been developed in many countries worldwide. Despite legal obligations, psychosocial risk assessments are rarely carried out in micro and small-sized enterprises (MSE). Even though those enterprises constitute a large proportion of the general workforce, this area remains largely untouched by research. In order to enable starting points for a greater dissemination in organizational practice, the present study explores barriers and drivers of psychosocial risk assessments in micro and small-sized enterprises.

**Methods:**

A total of 18 owners and managers from 15 micro and small-sized enterprises in North-Rhine Westphalia, Germany, were interviewed. The interviews were audio-recorded, transcribed, and analyzed. A qualitative approach was applied: Content analysis was used to analyze the data, using deductive as well as inductive coding techniques.

**Results:**

The following barriers emerged from the interviews: Negative PRA image, stigmatization of mental health, lacking acceptance of employees, fear of excessive authority interference, ignorance of PRA, not understanding the necessity, inappropriate approach, and limited resources. The identified drivers were: Easy access to PRA material, external support from experts, renaming the term “workplace risk assessment”, understanding content and benefit of PRA, simplify and revise PRA process, and noticeable consequences of PRA execution and non-execution. The results are comparable with those in larger companies. They emphasize the importance of mental health education, improving the process of psychosocial risk assessments, and the ongoing support in overcoming limited financial as well as human resources.

**Conclusions:**

To improve implementation of PRA in organizational practice, a simplification of the process is proposed, accompanied by information campaigns and an improvement in the health literacy of owners and managers of MSE. In view of the results, the full revision of the PRA approach should also be considered.

**Supplementary Information:**

The online version contains supplementary material available at 10.1186/s12889-021-11416-1.

## Background

Over the last years, research has demonstrated the negative impact of work stress – affecting physical [1–3] as well as mental health [4], all in all increasing the risk of mortality [5]. Hence, various efforts have been made to find ways for reducing the impact of work stress on an occupational level. Systematic approaches such as Workplace Risk Assessments (WRA) were developed as Occupational Health and Safety (OHS) interventions, and are nowadays required for employers in many countries – sometimes legally binding, sometimes not [6]. The European Union, for instance, has developed a framework for WRA in order to unify existing approaches across countries [7]. WRA with special focus on work stress, psychological hazards, and mental stress, − in the following referred to as “psychosocial stress” – is called Psychosocial Risk Assessment (PRA). Aside from work stress, psychosocial stress may result from emotional distress, job insecurity, or harassment at work [8]. Increased work demands, such as time pressure or high workload, can lead to unfavorable shifts in work-life balance [8, 9]. It is therefore important, to grasp a multitude of factors and their combined occurrence, in order to understand and improve the situation.

The PRA process includes identifying sources of stress in the working environment, developing and implementing an action plan, and subsequently evaluating the implemented measurements [10, 11]. The number of companies, which routinely execute PRA, is currently at a rather low level, especially if the whole process is considered [12]. In Germany, all companies are legally obliged to execute PRA in regular intervals (§5 ArbSchG, EU-OSHA, 2008), however many companies have not implemented PRA at all. It was recently reported that less than a quarter of all companies carry out PRA [13]. Micro and small-sized enterprises (MSE; 1–49 employees [14]) have the lowest implementation rate, and thus pose the group with the highest backlog in terms of psychosocial stress prevention [13]. In the year 2017, 41.5% of all employees in Germany were employed in MSE, resulting in almost half of the working population currently not included in PRA [15]. A trend that is also mirrored in other countries [16–18]. To improve this situation, it is important to investigate why PRA is not implemented in MSE. Previous research has identified barriers and drivers of PRA implementation, however not specifically in the MSE context. MSE is different to larger companies in several regards: it has typically less hierarchical structures, fewer resources and fewer trained personnel in terms of OHS management than larger companies [19]. This paper will explore barriers and drivers of PRA implementation exclusively in MSE from a qualitative interview perspective.

### Psychosocial risk assessments

The key elements of PRA are specified in the European framework for psychosocial risk management: defining a work population or a set of operations, assessing risks, designing and implementing actions for risk reduction and evaluating those actions [18, 20, 21]. The psychosocial risks addressed in PRA contain, for example, high workload, unfavorable working environments, low control, or conflicting interpersonal relationships. In Germany the process is specified by the Joint German Occupational Safety and Health Strategy [22] and comprises seven steps: (1) preparation of the whole process by defining the surveyed area, (2) measurement of psychosocial stress at work, (3) evaluation of psychosocial stress at work, (4) development and implementation of actions, (5) effectiveness control, (6) updating the process and maintaining it, and (7) documentation [11].

### Boundary conditions of PRA implementation

Despite the high proportion of employees in MSE, the attention of occupational health implementation has primarily focused on larger companies. Several studies summarize small and medium sized enterprises in one category (10 to 250 employees; SME; see Masi et al. [23] for an overview), thus ignoring micro enterprises with fewer than 10 employees. In the following section known barriers and drivers of OHS interventions are summarized.

In a qualitative interview study with SME owners and managers, most respondents do not see any obstacles of OHS improvements in their companies in general [24]. Other obstacles are costs, paperwork, other priorities, lack of training, time and staff. The most occurring barriers of OSH implementation identified are issues concerning stringent regulation, lack of resources and information, such as ineffective communication [23]. These findings go in line with barriers identified in studies primarily considering larger companies. Many owners and managers see no need for action at all, either because no hazards and risks are known, or because of an overall absence of major problems [25]. One major hindering factor is the lack of resources: It is reported that there is not enough time, staff, or money for WRA implementation [12, 18, 26–29]. Social aspects also play an important role in OSH implementation, like fear of non-acceptance, business climate concerns, difficulties with employee involvement, cultural gaps and hindering beliefs [12, 26, 28]. Other inhibiting factors refer to information, the lack of awareness, knowledge and expertise of owners and managers [12, 25]. Owners and managers are key to OHS implementation: if their approval and commitment is missing and if they have hindering beliefs or attitudes, the implementation and success of OHS interventions is endangered [26, 30]. In addition, actors at different organizational levels do not feel responsible, or their responsibility is not sufficiently delineated from other decision makers and authorities [30].

Several drivers for OSH implementation have been identified in previous research, although it is unclear whether these drivers may be transferred to MSE. Promoting factors often relate to financial consequences, such as reduction of insurance premiums, avoiding fines, and the motivating of maintaining or increasing productivity [25, 31]. An important factor before and during the implementation phase is the improved access to information: this includes knowledge availability, collaborations with networks and stakeholders, and improving the communication within the company [31, 32]. Commitments and legal requirements encourage the introduction and implementation phase by increasing the perception of obligation; for example having a company agreement has been identified as facilitating factor [25, 32]. Tracking the legal requirements, such as an inspection visit by OSH authorities, is increasing the likelihood of OHS implementation [13]. Previous studies have demonstrated that companies need support: Several aspects like external support of consultants, availability of occupational and safety specialist assistance, the use of supporting tools, as well as involving operational actors contribute to this factor [13, 31–33]. Furthermore, the presence of a works council and affiliations to the production sector is beneficial in this regard [13, 33]. Social aspects, for instance meeting employees’ expectations and maintaining the organization’s reputation also play an important role [25]. Having an OSH management system is strongly associated with PRA implementation [18]; moreover, concern for psychosocial issues and the request by employees facilitates PRA implementation. Sometimes critical events like high absenteeism rates or a decline in productivity, trigger PRA implementation [18].

To conclude, the main objective of this study is to generate knowledge on drivers and barriers of OSH implementation within MSE. As existing knowledge was neither exclusively generated within MSE context nor relates to the specific procedure of PRA, our study aims to qualitatively explore boundary conditions of PRA implementation by interviewing owners and managers from MSE.

## Methods

### Study design

The qualitative approach was chosen to generate detailed knowledge on PRA implementation in a specific population by interviewing decision-makers about their personal as well as practical experience on psychosocial stress prevention. The study was approved by the ethics committee of the Heinrich Heine University in Düsseldorf, Germany (reference number: 2019–460). Before the interviews, participants received brief information about the study, gave written consent and filled out a short demographic questionnaire.

### Study procedure

An interview guide was constructed ahead of the interviews in line with the following steps: First, as common procedure in qualitative content analysis [34], corresponding themes and interview questions were collected, verified by a group of experts in the field, then sorted, and finally sub-summarized. The interview guide was piloted by collecting written feedback from health experts and by conducting two pretests. The full interview guide can be found in additional file 1. The semi-structured approach allowed us to ask follow-up questions in response to participant’s answers. This flexibility to change the order of questions if, for example, participants answered some questions that were not to be asked until a later point helped to incorporate the natural flow of the conversation. Our interest in the subjective point of view of participants, as well as their practical experience, was emphasised. The interviews lasted between 30 and 90 min and consisted of four main parts: (1) introduction, (2) view on psychosocial stress, (3) psychosocial risk assessments, and (4) health measures. The different length of the interviews came from the different prior experience with psychosocial stress and PRA as well as the time and willingness to talk about it. The present study will focus on analysing part (3) and (4); analysing (2) is part of another study (publication in progress).

### Research setting

The present study was conducted in the context of a large research project. The majority of the 18 interviews were personal interviews (*N* = 13) at the workplace of the interviewees, while five interviews were done via phone call. The participating companies were located is the district of North Rhine Westphalia in Germany; the interviews were conducted in German. All interviews were conducted by the first author. The interviewer and study participants have had no contact up to this point, other than to arrange the interviews. The participants were recruited through networks of members of the research project’s advisory board. After it became apparent that it was difficult to recruit MSE owners and managers, we additionally recruited respondents from a business register that lists companies in the study area. The data was gathered by conducting semi-structured qualitative interviews between July 2019 and April 2020. Four interviews were carried out during the Corona pandemic; the current situation was addressed but the questions referred to the working conditions before the pandemic.

### Data collection

The interviews were audio-recorded, subsequently transcribed, and anonymised. The applied transcription rules followed common transcription guidelines [35]. The consolidated criteria for reporting qualitative studies [36] are reported in the additional file 2. The transcripts were reviewed by the first author to verify the correct transcription. The participants voiced no interest in reviewing the transcripts (but in the overall result of the study), so the individual transcripts were not returned. The data was gradually analysed; as soon as content saturation occurred, recruitment for the interviews was terminated. We followed a data-oriented saturation perspective that assumes saturation when new data only repeats what was expressed in previous data [37].

### Participants and company characteristics

A total of 18 owners and managers from 15 MSE in Germany participated in the study. In some cases, more than one person per company participated in the qualitative interview due to shared responsibilities. Selection criteria were willingness to participate, an interest in talking about health issues concerning working conditions, and considering the implementation of PRA in their company. The age of the MSE owners and managers ranged from 27 to 63 years (*M* = 49.3, *SD* = 9.0). Most of MSE owners and managers were male (88.9%). Half of them had practical apprentice training (*N* = 9), while the other half finished their education with some form of university degree (*N* = 9). Work experience varied; on average they had 15.6 years leadership experience (*SD* = 10.5; range 1–36 years), and were employed at the present company for 13.8 years (*SD* = 10.9). See Table 1 for the company characteristics.

**Table 1:** Company characteristics

	Micro enterprises	Small enterprises	Total	Female ratio
Service Sector^a^	4	5	9	48.7%
Craft Sector	2	4	6	20.4%
**Total**	6	9	15	37.4%
Number of employees (SD)	6.3 (2.3) Range: 3–9	21.3 (11.5) Range: 12–45	15.3 (11.7)	
Mean age of employees (SD)	35.6 (6.9) years Range: 28–45.7	41.7 (5.4) years Range: 35–50	39.3 (6.6) years	

^a^ including one health sector company

### Data analysis

The data was analysed using the MaxQDA 2018 software. Established qualitative content analysis was applied [38–40]. Content analysis is a research method that enables researchers to retrieve replicable and valid interferences from data by forming categories that reflect the structure and describe the content of the qualitative data [41]. The data analysis applied can be differentiated in two steps: first, based on literature review and our research questions, a category system was developed (deductive approach). Secondly, the category system was revised, and new categories were added (inductive approach). Subsequently, the category system and the coding itself was reviewed by two independent researchers. The reviewer consensus rate was at 98.5% by the first and 98.4% by the second reviewer. Finally, the category system was further refined based on the feedback in a second round of coding; however, only few adaptions were necessary. The hierarchy of the final category system consisted of three levels: the main research questions serving as main categories, while the generic categories describe different components, which are further differentiated in sub-categories. The two main categories are defined as follows: Barriers are detaining, inhibiting factors and, in contrast, drivers are promoting, facilitating factors of the PRA process, the implementation and its circumstances based on the perception of MSE owners and managers. The generic categories, e.g. barriers and drivers, are explained in detail in the results.

## Results

**Fig. 1:**
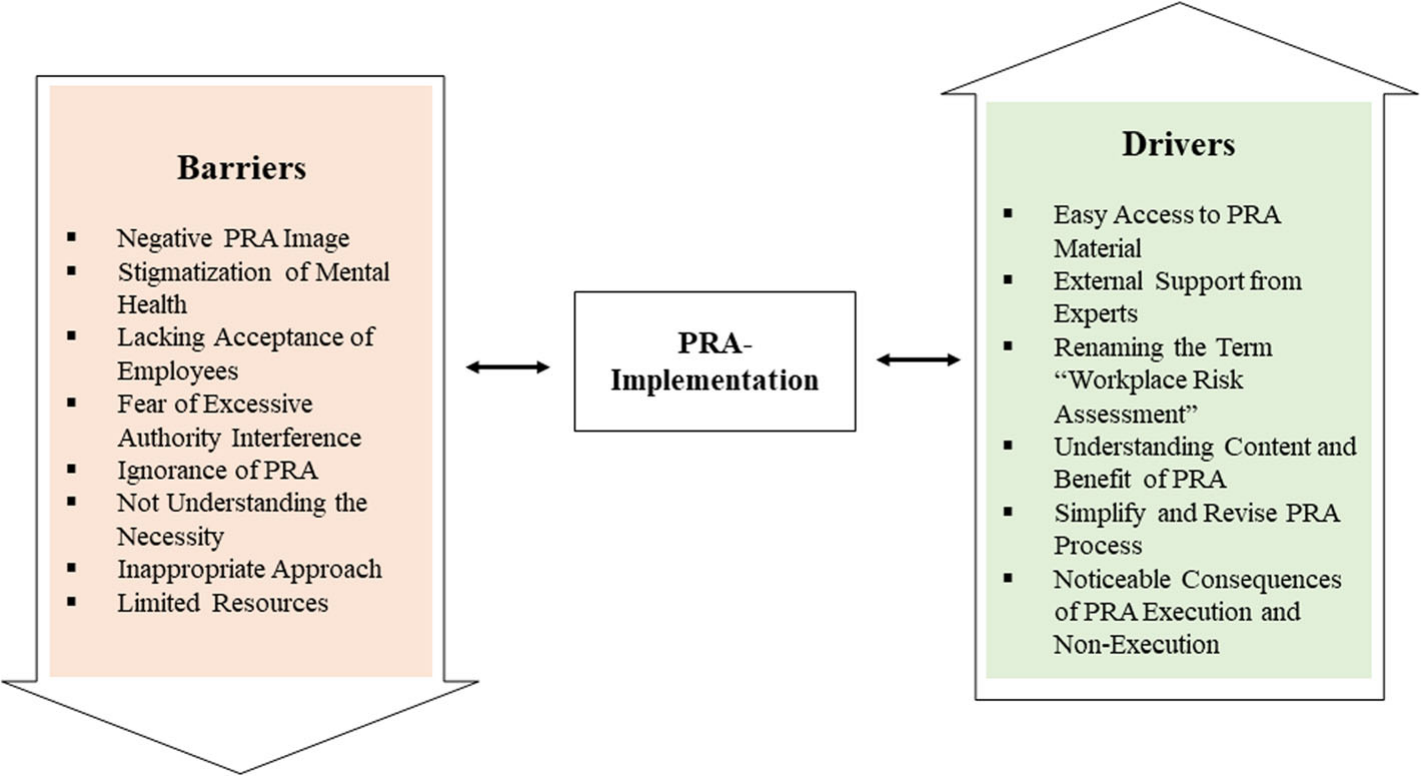
Graphic illustration of barriers and drivers of PRA implementation

In total, eight barriers and six drivers of PRA implementation in MSE emerged from the data; see Fig. 1 for a graphic illustration. The Results section is supported by literal quotes (LQ) in the text that refer to the corresponding quotes listed in additional file 3.

### Barriers

#### Negative PRA image

Some MSE owners and managers did not see the potential of PRA for health promotion. In addition, some of them associated it with an unnecessary burden or an inconvenient obligation. The PRA execution was perceived as another item on the long list of administrative tasks required by the authorities. As with other administrative tasks, it was perceived as something that needs to be done because it is required rather than because it is beneficial. The perception as a boring and tiresome duty lead to low motivation and a negligent PRA implementation.

*“So, I’ve worked at several companies and most of them see it as a necessary obligation. And I also know that many [ …] don’t go to the facility at all, the thing is filled out at the desk, then it was done and basta.”*

#### Stigmatization of mental health

Many respondents reported stigmatization of psyche and mental health, particularly in the craft sector and in male-dominated work environments. According to MSE owners and managers, mental stress is associated with weakness, and as a result, means admitting that one is weak.

*“Mental stress, stress, is always a bit of weakness, yes. That’s why [ …] everyone whines, I have too much to do, nobody will admit it without further ado, yes, I’m about to go to therapy now.”*

Respondents did not necessarily agree with this themselves, but they feared that others might think so. The severity of psychosocial stress is still underestimated because the effects were more difficult to grasp compared to physical illness (additional file 3; LQ1). Another aspect was the association of mental health with something private, which led to the attitude that everybody has to find solutions for themselves (LQ2).

#### Lacking acceptance of employees

One reason for not implementing health programmes is reflected in the assumption of the MSE owners and managers, that their employees will not support it. As described in the previous paragraph, psychosocial stress was often seen as a rather private matter. Therefore, tackling this issue would mean an interference with their employees’ privacy.

*“I think there is an inhibition threshold for some employees to do this. They would, if they have the need for support - I think - would perhaps prefer to find it privately. Outside the company”.*

As a result, the potential benefit of PRA seemed questionable, because this would exceed their authority as owners and managers. Several MSE owners and managers voiced these concerns (LQ3). Another point was that stress can have several different causes, some of which are rooted in personal life. As a consequence, the owners and managers did not really believe they were responsible for fixing this, but shifted the need for action, at least in part, to the employees (LQ4).

#### Fear of excessive authority interference

MSE owners and managers feared that PRA could be overly regulated by the authorities. For example, outside organizations - like employers’ liability insurance associations - were generally associated with regulation and sanction. Several MSE owners and managers had the attitude that the less the employers’ liability insurance association knew about their work environment, the better. Although MSE owners and managers thought they were not (knowingly) doing anything wrong, they feared the discovery of something that would get them in trouble or cause even more work.

*“On the other hand, I admit that as an entrepreneur you are immediately afraid that this will be like with the new General Data Protection Regulation. If these regulations hinder you in such a way that one can no longer do their work, then it was too much advice from outside.”*

MSE owners and managers highly valued their independence. External interference and regulation could therefore jeopardize independence in that they would rather not do anything that could add to their workload or result in them losing control of the company.

#### Ignorance of PRA

Another reason for not conducting PRA is that MSE owners and managers simply had not heard of it, *“we had no idea that there was such a thing and what it included. We still don’t really have it. So that’s why we are very happy that you are there.”* Several MSE owners and managers admitted that they had neither knowledge of the term itself, the content, nor the legal requirements – summarizing, *“absolute ignorance”*. The legal requirements were broadly unknown, *“up until our phone call I was unaware that it was somehow a legal requirement*”. However, PRA ignorance did not mean that the subject was ignored. Most MSE owners and managers reported solutions for psychosocial stress on an individual, informal level – skipping the systematic approach of PRA but considering the individual needs of their employees (LQ5).

#### Not understanding the necessity

We found that one major PRA barrier was the lack of understanding of the need to conduct PRA and the potential benefits of health prevention. We identified several aspects contributing to this factor: First, the need for the implementation was low, because the underlying reasons as well as the benefits were unclear to MSE owners and managers.

*“And have we needed it so far? No. Have we managed without it so far? Yes. OK. And just when this is required by law, I believe that many entrepreneurs are not always willing to implement this if it does not seem logical to them.”*

Second, the content of PRA was seen in part as common sense and, therefore, perceived as a redundant process (LQ6). Third, MSE owner managers saw no need as there was no significant psychosocial stress that needed special attention (LQ7). Fourth, MSE owners and managers had divergent expectations of the PRA concept. For example, the lack of reoccurring risks was seen as a reason not to implement PRA. The occurring risks were not considered significant enough to justify the effort of PRA implementation (LQ 8). Fifth, the belief of having self-reliant employees attenuated the necessity of PRA execution, because it made PRA partially redundant. MSE owners and managers trusted that their employees would call in if problems arose, including issues with psychosocial stress, or that they would notice it themselves. Many companies cultivated an open discussion culture and individually solved problems as they came along (LQ9, LQ10).

#### Inappropriate approach

Another important barrier lies within the method itself: MSE owners and managers questioned whether PRA is the appropriate approach for MSE. One critical aspect is that they felt that the approach was not tailored to their profession.

*“I also found that these questionnaires are really focused on the craft industry [ …]*. *And I think you just have to differentiate that. So, we have redesigned many, many things ourselves, also with regard to questionnaires. Simply because you have to see it in a job-related way.”*

Furthermore, the size of the company plays an important role: the smaller the company the less sense was seen in PRA. Even the difference between three and ten employees was perceived to make a difference (LQ11, LQ12). As already mentioned, a systematic approach was criticised because it failed to consider the individuality of MSE and did not adapt to changing work conditions. This also resulted in MSE owners and managers questioning, if PRA is the correct approach for MSE altogether (LQ13). Criticism was also voiced about the PRA process itself: The method cannot guarantee meaningful results, for example when employees fill out the PRA questionnaires mechanically without really considering the questions. The process was also seen as complicated, especially for the micro enterprises, since the required effort did not seem to outweigh its benefits (LQ14, LQ15). MSE owners and managers also voiced suggestions for alternate approaches, for example a shorter, more informal approach (LQ16).

#### Limited resources

We found a key factor in why MSE owners and managers rarely execute PRA to be the lack of resources, such as time, finances, and trained personnel. MSE often neither have personnel with Health and Safety expertise, including psychosocial stress, nor the time to do so.

*“And it is not really the case with the size of the company that you have someone from Human Resources sitting there who takes care of such things”*.

MSE owners and managers felt underqualified themselves and did not have the external support that they would need. Since the benefit of PRA was often unclear, the motivation to invest resources was low*.* Quite often, day-to-day business consumed most of the available resources; not executing PRA was down to having no extra capacity for it (LQ17, LQ18). Limited resources were certainly a major contributing factor – however, sometimes it was rather a question of resource distribution and prioritizing (LQ19).

### Drivers

#### Easy access to PRA material

One of the first steps to increase PRA implementation, is to make PRA material easily accessible. MSE owners and managers described not knowing how to find information about PRA implementation and execution. Since there are not only different PRA variations but also different providers, it takes effort to find information and to single out the most suitable solution. Effort, that many were not willing to make.

*“If you don’t get it sent, you don’t sit down and search the websites yourself. Where could I find something? Do I go to a professional association, do I go to a university clinic, do I go to students, do I go there? It’s missing.”*

Furthermore, as there are often many other pressing issues for owners and managers, a more proactive approach was suggested.

#### External support from experts

MSE owners and managers saw the support of external experts as an important contributing factor to the successful PRA execution. Some of them simply felt like they did not have enough expertise dealing with the topic of psychosocial stress and health interventions: *“I think it’s great when you can outsource such topics, to people who also know what the consequences are”.* They also saw several advantages in a distant point of view.

*“It has a great advantage if external people come in, they are not blind to the company. First, they see it with different eyes. Especially when they don’t work here or don’t work in this area, the risk potential simply looks very different. We would say it has always been like this, it is normal, it is part of the work.”*

In the MSE owners’ and managers’ opinion, a neutral point of view – as is the case with outside experts – would also increase the employees’ acceptance of the intervention, because the employees can speak more freely and are less worried that their critical input can be used against them afterwards (*“So I think a neutral person like you definitely gets more truth told than I do”*). From their perspective, it would also circumvent the fear of the employees that they are using the intervention to push their own agenda (LQ20).

#### Renaming the term “Psychosocial Risk Assessment”

What is “Psychosocial Risk Assessment”? How do you understand the term, what does it mean to you? The vast majority of MSE owners and managers answered this question with a quite literal description of the term – assessing risks. Nobody mentioned an underlying process, an action plan, or the evaluation phase. This result can partially be explained by the fact that the general knowledge about PRA was rather sparse. However, the term is not self-explanatory – it merely describes one step of the process rather than the whole process. The statements in this aspect were quite clear:

- *(A) “Maybe I am too much of a lawyer again, but an assessment is close to judgment and a judgment has a point at the end. It is exactly the opposite of a cycle.”*
- *(B) “In that case, even if I had known that a risk assessment had to take place, I would have imagined something wrong.”*

Some MSE owners and managers even had negative associations with the term (LQ21).

#### Understanding content and benefit of PRA

A central aspect in the interviews was the lack of understanding about the content and the benefit of the PRA. The psychosocial aspect of the PRA was broadly unknown in most companies in this study; in the craft sector the Risk Assessment for physical risks was a little better known. To implement PRA in their company, MSE owners and managers wanted to be convinced of the benefits, before they invest precious resources (LQ22). Several owners and managers emphasized the importance of information and sensitization as key factors for the adoption (LQ23). In addition, personal concern was another aspect in adopting health prevention programmes. Unless owners and managers have had issues with mental health either themselves or somebody in the company has had it, the incident rate and thus importance of stress prevention was underestimated (LQ24). Several owners and managers described difficulties referring to the broad subject of psyche and mental health (LQ25). Another potential solution that MSE owners and managers suggested, was to break the PRA benefits down to numbers.

*“I can show them how effective - based on numbers maybe - how effective an employee is who feels good in relation to someone who is forced to do his service, who is forced to work overtime, I think you can see clear performance curves there.”*

The reasoning behind PRA was rated higher than the obligations by the law (LQ26). According to MSE owners and managers, the readiness of implementation will be encouraged, if the field of psychosocial stress is more relatable and the personal relevance is highlighted.

#### Simplify and revise PRA process

As described in the introduction, the PRA process in Germany in its original form is comprised of seven steps. Especially for MSE, this created the appearance of a rather extensive procedure, which on its own represents an obstacle for the implementation. The MSE owners and managers in this study wished for a simplified process that can be easily understood, and that can be implemented without having to invest many resources. As a result, the process should be modified to a simplified version, for example a reduction to key factors that indicate possible critical aspects of psychological stress (LQ27). Moreover, MSE owners and managers doubted if the methods of PRA are suited the structures of MSE. Methods like employee surveys were critically viewed because they neither address the individual properties of MSE nor take their specific industry into account (LQ28).

#### Noticeable consequences of PRA execution and non-execution

Having a widely unenforced law did not cause enough need for action. To increase the motivation for action, PRA execution should be rewarded and non-execution more severely sanctioned. Some MSE owners and managers would prefer benefits, such as bonus programmes, financial support, or Health Certificates instead of penalties.

*“So actually, in my opinion it is the reward principle, “here we have a program, you will then receive a certificate, can advertise that you take care of health protection”. […] This will appeal to companies rather than penalties.”*

Others pointed out that having such a law only makes sense, if obeying is enforced and ultimately punished if it is violated (LQ29).

### Insights into operational practice

Overall, PRA was not very well known and not often applied in MSE. This, however, does not imply that stress prevention or mental health issues were generally neglected. Typically, MSE owners and managers dealt with these issues on an individual basis. Many companies maintained an open discussion culture, and knew each other well within the company. The lack of hierarchical structures can be an advantage, as measures and change processes can be kept short and implemented quickly. Reported measures are, for example, making changes in the work schedule, changing the workload distribution, giving extra time off, or the acquisition of technical equipment. However, the care of the MSE owners and managers primarily depended on their own knowledge and initiative and measures referred to psychical rather than mental health. The measures were therefore often behaviour measures instead of changing work conditions.

## Discussion

The aim of the study was to identify barriers and drivers for the PRA implementation exclusively in MSE from a qualitative interview perspective. A total of 18 MSE owners and managers from 15 MSE participated in the study. The identified barriers and drivers of PRA in MSE are in line with previous research [17, 24–26]: It shows that factors like lack of resources (due to not enough staff, money or time), insufficient training, fear of non-acceptance, other priorities and no general need for PRA hinder its implementation [23]. On the other hand, the improved access to information, external support by experts and financial consequences, for example fines, are drivers of PRA implementation that we also found in our study [31]. However, our findings extend known factors; the new aspects of PRA implementation in MSE are discussed in detail in the following sections. Many factors can be attributed to either the PRA process or mental health and health competence. We will therefore focus on these two main aspects in the following sections.

### Revision of the PRA process

There were several issues directly related to PRA implementation: First, PRA was not well known to MSE owners and managers, neither the term nor the process required. This resulted in an incomplete or no PRA implementation. It also often coincided with MSE owners and managers not understanding the need for PRA. Second, if PRA was known, it did not have a reputation for being a beneficial process but rather a duty required by the authorities. Both, the negative PRA image as well as the fear of excessive authority interference, reduced the attractiveness of PRA and consequently inhibited PRA initiation. Third, MSE owners and managers questioned, if systematic approaches such as PRA are appropriate in MSE. The process and its methods were considered complicated and not well suited to the very individual MSE environment. One of the major barriers for PRA implementation in MSE was the lack of resources, such as time, money, and trained personnel. If MSE owners and managers had the opinion that there was no financial or time capacity for OHS, the management of the operational business was prioritized.

To improve the PRA process itself, several adjustments would be beneficial: PRA could be made more attractive, by explaining its benefits better and to emphasise its positive aspects [42]. MSE owners and managers suggested renaming the process to raise the understanding and attractiveness of PRA. Furthermore, the PRA process should be simplified, but still consider specific different settings and characteristics of MSE [43]. This requires a process, that offers individual options and is flexible without being overly complicated. In fact, the PRA fulfils these points relatively well, but due to the widespread distribution of PRA-material and the complex content, it is predominantly the group of health experts who know how to handle it [11, 44]. Considering the lack of resources in MSE, it seems unrealistic to expect MSE owners and managers to either independently execute PRA or to spend extra resources on it. One solution could be to support MSE owners and managers with the PRA execution for example by outsourcing the execution to external, independent entities. To make it easier and to take the financial background of MSE into account, financial incentives, bonus programs or other rewards could be used for additional motivation. Another option is to change the PRA method, for example away from surveys towards an informal, communicative approach [21]. Finally, as the implementation or non-execution of PRA had no consequences in most companies, it was suggested that the execution of PRA should either be rewarded or more severely sanctioned by the authorities. Positive reinforcement as well as positive psychology have both proven to be powerful motivational drivers of behaviour change and change processes [45, 46]. Monetary benefits are certainly one solution, but implementing PRA can have many positive effects that could be highlighted, for example: raising employee loyalty, improving work processes, saving money through less lost work due to ineffective work or illness, becoming more attractive as an employer, and preventing illness caused by work. The results of the study demonstrate the need for a simplified, easy-to-use, self-explaining approach that is tailored to the needs and specifications of MSE. Research on finding solutions for this need has been recognized; psychosocial stress in the context of small companies is given more attention [19, 47, 48] and models for interventions are being developed [49]. New ideas, such as Online Interventions, eHealth, and Apps are being explored [50].

### Mental health and health competence

MSE owners and managers had reservations about addressing psychological issues in the work environment and feared that their employees would disapprove of it. The stigmatization of mental health and lacking employees’ acceptance created an unfavourable framework for working conditions that reduced the motivation of dealing with this issue and often hindered the PRA process before it had even started. The general acceptance and openness of psychological topics is an issue that is not specific to OHS interventions but is observed in other areas as well. What is often referred to as Mental Health Stigma is difficult to solve, because it requires a change in public attitudes, which takes time and involves many players [51, 52]. Nevertheless, in MSE the Mental Health Stigma is of particular importance, as relationships in MSE tend to be more informal and personal than in larger companies [53], thus protecting one’s privacy is more of a challenge. Moreover, as there are usually hardly any independent instances within the company, raising this topic could be difficult. As a result, it can be assumed that the situation in MSE is exacerbated compared to larger firms with similar issues.

Given the barriers outlined before, increasing knowledge about mental health and PRA is a central aspect of improving PRA implementation. This includes making it easier to access PRA material, making PRA more noticeable, and explaining the content and benefits of it to MSE owners and managers and their employees in more detail. Although some aspects of mental illness are without doubt private, unfavourable work conditions as possible cause are not. It is therefore necessary, to outline the differences and improve the general understanding of mental health. Nevertheless, privacy should be given special consideration when choosing the approach. The attribution of stress being individual and something private, holds the danger of overlooking, for example bad working conditions as a major contributing factor, and, therefore reduces the likelihood of making any changes in the working environment. The diffusion of responsibility should be avoided, for example by identifying those responsible and training them. Establishing accountability has previously been found to be beneficial for OHS implementation [24]. The importance of understanding mental health to facilitate favourable, preventive health behaviour is backed by several theoretical frameworks, for example the Health Belief Model [54]. In short, it constitutes that antecedents of action are largely determined by the perceived benefits and the expected costs. Thus, it predicts that the likelihood of action would increase, if the expected benefits were raised, and the costs were reduced.

MSE also have advantages compared to larger companies: the lack of health competence might be attenuated by the high social responsibility and social cohesion observed in smaller companies [55, 56]. However, the effect is ambiguous and depends on the specific context.

### Strength and limitations

The qualitative content analysis applied in this study allowed us to gain new insights into the mindset of MSE owners and managers and to better understand the situation in practice. These findings are valuable for the further development of systematic stress prevention, and can thus contribute to the goal of increasing the implementation of PRA in MSE. As the study is based on qualitative data, the general limitations of qualitative research also apply to this study: The qualitative data can be influenced by the interviewer and the evaluator; therefore, the subjectivity of the interpretation cannot be ruled out. To remedy this, the results were assessed by two independent researchers, which had a very high consensus rate. Furthermore, the many verbatim quotations in the additional files are intended to enable an independent assessment to the reader and thus increase transparency. Due to the voluntary participation, selection bias cannot be ruled out. It can be assumed that participants were generally open to health issues and were willing to disclose information about their company. A mixed-method design could be used to validate the results on a quantitative level within a larger, representative sample. The findings of this study could be further refined by presenting the findings to OHS experts and compare the results. Moreover, to get a more detailed insight, the steps of the PRA process could be analysed in detail [57].

## Conclusions

Based on qualitative interviews with MSE owners and managers, it was revealed that systematic approaches to prevent psychosocial stress are too often amiss in MSE. Due to a negative image of PRA and general mental health stigmatization, MSE owners and managers should be motivated for example by demonstrating the need for PRA and by providing information campaigns carried out by professional associations, guild health insurances, or other OHS providers. As MSE owners and managers may neither have the required background knowledge nor the time to acquire it, experts can either assist with the internal PRA implementation or handle the entire process. Simplified solutions for psychosocial stress prevention should be developed that shorten familiarization with the topic. Especially for micro enterprises the whole process is unwieldy to manage, thus, OHS experts, and the Legislative in particular, should also consider the complete overhaul of the approach. This article offers many starting points for improving stress prevention and PRA implementation in MSE. The emergence of the Corona virus and its consequences has further demonstrated the need for alternative approaches that can provide stress prevention also from the distance, for example digital prevention programmes.

## Supplementary Information

**Additional file 1.** Interview guide with MSE owners and managers

**Additional file 2.** Consolidated criteria for reporting qualitative studies (COREQ) checklist

**Additional file 3.** Additional verbatim quotations to provide insights into the mindset and attitudes of MSE owners and managers.

## Data Availability

The category system is available from the corresponding author on reasonable request.

## Abbreviations

LQ: Literal Quote
MSE: Micro and Small-sized Enterprises
OHS: Occupational Health and Safety
PRA: Psychosocial Risk Assessments
WRA: Workplace Risk Assessments
