# Supplementary material for: Barriers and drivers of psychosocial risk assessments in German micro and small-sized enterprises: a qualitative study with owners and managers
Source: BMC Public Health. 2021 Jul 12;21:1376. doi: 10.1186/s12889-021-11416-1 (PMC8273035; doi:10.1186/s12889-021-11416-1)
Supplement: Supplementary file 1 — Additional file 1. Interview guide with MSE owners and managers [file 12889_2021_11416_MOESM1_ESM.pdf]

Additional file 1. Interview guide with MSE owner-managers

I. Introduction

- Thank you for your willingness to talk
- Introduction of the interviewer
- The conversation today will be about health and working conditions in your company

Method:

- The interviews will last about 45 minutes
- I've brought a few questions here that I'll be asking you one by one. Since I don't know all the questions by heart, I will sometimes read out questions or look at my documents and make notes - don't let it confuse you.
- After the interview part, we are happy to talk about the further course of the project.
- One important note in advance: There are no right or wrong answers, we are primarily interested in your point of view.
- I will keep an eye on the time so that we roughly stay within the time frame.

Confidentiality, data protection and declaration of consent

- In order to be able to analyze the content of the interview afterwards, we will record the conversation *[show device!]*
- When written down, the data is anonymized, i.e. all personal information, such as names, are made unrecognizable. We already sent you a few documents in advance (the questionnaire about yourself and your company, the participant information, and the declaration of consent). Have you read and signed the documents, or do you have any questions? *[accept signed documents, otherwise go through together and have them signed]*
- Do you have any questions before we begin?

[Begin audio-recording]

II. Interview

1. *[Optional]:* What does your company specialize in?
2. How did you hear about us?
3. What made you decide to take part in the study?

III. General health

As already mentioned, our conversation will be about health in your company, so ...

4. To begin with, please tell us in general how the topic of health is dealt with in your company?

→ Health is a topic	→ Health is no topic
a) Is health important to you?<br>b) What are the topics?<br>c) How is it talked about? [Employees among each other?]<br>d) Do your employees sometimes also talk to you about health issues?<br>e) In what context is it discussed?<br>f) How do you find out about health?	a) a) Why could it be that health is not an issue?<br>b) b) What are important issues in your company?<br>c) c) Do you think health is important?

[...]

V. Psychosocial Risk Assessment

As you have probably already noticed in advance, our project is also about risk assessments.

8. Are you familiar with risk assessment? Do you understand what is meant by that?

   a. Yes → Please briefly explain what it means to you.

   b. No → In risk assessments, stresses and dangers that can arise in the course of employment are analyzed. If unfavorable conditions are found, measures for improvement are developed. After the measures have been implemented, an evaluation is conducted to see whether the measures have worked. A risk assessment is a process aimed at reducing stress and promoting good aspects of work. So, it's not just about avoiding dangers, but also about for example optimizing work processes and strengthening resources.

9. Are risk assessments or something similar carried out in your company (e.g. risk assessment for maternity leave)?

   a. If so, which ones and how often?

10. Have you ever heard of the psychosocial risk assessment and if so, what do you mean by it? *[If description is not sufficient, explain PRA]*

a. What is your association with it?

11. Do you carry out risk assessments for psychosocial stress in your company?

→ Yes	→ No
a) How do you do that exactly?<br>b) Why are you doing a PRA?<br>c) How often and on what occasions is the psychosocial risk assessment carried out?<br>d) To what extent are the psychosocial risk assessments of and the general risk assessment combined? *[i.e. are there shared responsibilities, structures, and experts?]*<br>e) To what extent do you consider the frequency and scope to be appropriate?<br>f) *[if irregular intervals]* What is preventing you from carrying out the psychosocial risk assessment regularly / more frequently? *[which barriers]*<br>g) What would make it easier to carry out psychosocial risk assessments?	a) Why have you not carried out a psychosocial risk assessment so far? *[Barriers, obstacles, lack of resources or qualifications]*<br>b) What could a practicable approach to health improvement look like?

12. Do you consider a risk assessment of psychological stress to be sensible in principle?

13. What could companies that have not yet carried out risk assessments help to do this?

[...]

VIII. Ending

17. Have we forgotten something that you would like to mention?

Thank you, we have now come to the end of the interview.

[Stop recording]
