# Supplementary material for: Barriers and drivers of psychosocial risk assessments in German micro and small-sized enterprises: a qualitative study with owners and managers
Source: BMC Public Health. 2021 Jul 12;21:1376. doi: 10.1186/s12889-021-11416-1 (PMC8273035; doi:10.1186/s12889-021-11416-1)
Supplement: Supplementary file 2 — Additional file 2. Consolidated criteria for reporting qualitative studies (COREQ) checklist [file 12889_2021_11416_MOESM2_ESM.pdf]

Additional file 2. Consolidated criteria for reporting qualitative studies (COREQ) checklist[1]

**No. Item**	**Guide Questions/Description**	**Reported on Page #**
**Domain 1: Research Team and Reflexivity**		
Personal Characteristics		
1. Interviewer/facilitator	Which author/s conducted the interview or focus group?	VP
2. Credentials	What were the researcher's credentials? e.g. PhD, MD	Title page
3. Occupation	What was their occupation at the time of the study?	Research assistant
4. Gender	Was the researcher male or female?	female
5. Experience and training	What experience or training did the researcher have?	University degree, practical experience
Relationship with Participants		
6. Relationship established	Was a relationship established prior to study commencement?	2.3 Research Setting
7. Participant knowledge of the interviewer	What did the participants know about the researcher? e.g. personal goals, reasons for doing the research	2.3 Research Setting
8. Interviewer characteristics	What characteristics were reported about the interviewer/facilitator? e.g. bias, assumptions, reasons and interests in the research topic	N/A
**Domain 2: Study Design**		
Theoretical Framework		
9. Methodological orientation and theory	What methodological orientation was stated to underpin the study? e.g. grounded theory, discourse analysis, ethnography, phenomenology, content analysis	2.6 Data analysis
10. Sampling	How were participants selected? e.g. purposive, convenience, consecutive, snowball	2.3 Research Setting
11. Method of approach	How were participants approached? e.g. face-to-face, telephone, mail, email	2.3 Research Setting
12. Sample size	How many participants were in the study?	2.5 Participants and company characteristics
13. Non-participation	How many people refused to participate or dropped out? Reasons?	None
Setting		
14. Setting of data collection	Where was the data collected? e.g. home, clinic, workplace	2.3 Research Setting
15. Presence of non-participants	Was anyone else present besides the participants and researchers?	2.3 Research Setting

16. Description of sample	What are the important characteristics of the sample? e.g. demographic data, date	2.5 Participants and company characteristics
Data Collection		
17. Interview guide	Were questions, prompts, guides provided by the authors? Was it pilot tested?	2.2 Study procedure
18. Repeat interviews	Were repeat interviews carried out? If yes, how many?	No
19. Audio/visual recording	Did the research use audio or visual recording to collect the data?	2.4 Data collection
20. Field notes	Were field notes made during and/or after the interview or focus group	2.3 Research setting
21. Duration	What was the duration of the interviews or focus group?	2.2 Study procedure
22. Data saturation	Was data saturation discussed?	2.4 Data collection
23. Transcripts returned	Were transcripts returned to participants for comment and/or correction?	No; 2.4 Data collection
**Domain 3: Analysis and Findings**		
Data Analysis		
24. Number of data coders	How many data coders coded the data?	2.6 Data analysis
25. Description of the coding tree	Did authors provide a description of the coding tree?	2.6 Data analysis
26. Derivation of themes	Were themes identified in advance or derived from the data?	2.6 Data analysis
27. Software	What software, if applicable, was used to manage the data?	2.6 Data analysis
28. Participant checking	Did participants provide feedback on the findings?	No
Reporting		
29. Quotations presented	Were participant quotations presented to illustrate the themes/findings? Was each quotation identified? e.g. participant number	Results, all paragraphs; Supplementary Material
30. Data and findings consistent	Was there consistency between the data presented and the findings?	Discussion, first paragraph
31. Clarity of major themes	Were major themes clearly presented in the findings?	Results, Figure 1
32. Clarity of minor themes	Is there a description of diverse cases or discussion of minor themes?	Results, paragraph 1 -14: descriptions of minor themes
[1.] Tong A, Sainsbury P, Craig J. Consolidated criteria for reporting qualitative research (COREQ): a 32-item checklist for interviews and focus groups. *Int J Qual Health Care* 2007; 19(6):349 – 357		
