# Supplementary material for: Barriers and drivers of psychosocial risk assessments in German micro and small-sized enterprises: a qualitative study with owners and managers
Source: BMC Public Health. 2021 Jul 12;21:1376. doi: 10.1186/s12889-021-11416-1 (PMC8273035; doi:10.1186/s12889-021-11416-1)
Supplement: Supplementary file 3 — Additional file 3. Additional verbatim quotations to provide insights into the mindset and attitudes of MSE owners and managers. [file 12889_2021_11416_MOESM3_ESM.pdf]

Additional file 3. Additional verbatim quotations to provide insights into the mindset and attitudes of MSE owners and managers.

(LQ1)	*"If someone has a burnout or other head-related illnesses, I think the craftsmen are less willing to talk about it. I speak for the craftsmen, I think. It's not for them. You can't fix that. With the leg I can put a splint on it, put a wooden board on it and then it's fine. And I think the uncertainty to deal with it is more evident. And this also makes the obstacle of dealing with it more difficult."*
(LQ2)	*"Psyche was always something where you are convinced, when it's like, someone just doesn't get along with work or he has his own problems with the income and is now not at it, he has to regulate himself."*
(LQ3)	*"Honestly, I am sceptical, because there are certainly always some reservations on the part of the employees, perhaps one of the employees: "Would I like that within the scope of the company, I would like that my colleagues might notice it or my boss would somehow know that I might have a problem with strains of whatever kind?""*
(LQ4)	*"I believe that, on the one hand, this could be a bit too much of an intrusion into the privacy of the employees, because the psychological stress or the risk at work, if I ask around, one or the other employee could of course also take it badly, something like - now he also takes care of these things, but it's my business, how I deal with it. It can also be the consequence of personal problems at work. And there is the question, is it something that concerns the employer, or is it something that, if he interferes, has rather negative consequences. So, some employees always say, "that's my cup of tea"."*
(LQ5)	*"Yes, as I said, I didn't know anything about it up until now and didn't do anything by the book. One could say, as an entrepreneur I have done a risk exposure in the sense that I have at least tried to analyse sources of fire of the threat to our mental stress, to identify them and to work on them, right? So, I*

	mean, just because I haven't used models that are set according to any schema does not mean that I have not dealt with the topic. I would put it that way."
(LQ6)	*"I always find it interesting that you have to press it in a list so abstractly in order to create a learning effect. Because, I'm actually convinced that the person with the evolution behind it should actually be able to hold the finger on the nail and hit the hammer on it / so common sense actually says that it is a bad position for my thumb, please hold on to something else or something. So, I believe that common sense could replace many risk analyses. But I also know that it doesn't work in some places, probably."*
(LQ7)	*"No, I don't think I have any stress in my company. I probably do have it, but at least not in a way that it stands out or that it stands out negatively. So, stress / can also be that there is a lot to do and the day is a little busy. That different things in a day don't work as you had imagined. There we are again at a point where I say, "What is stress?"."*
(LQ8)	*"In other areas I see no foreseeable recurring risk factors that recur at least so frequently that it is economically understandable that I now think about how I can manage this risk as an entrepreneur. So, the risk, my contact person is a complete idiot and really gets on my nerves, so in the truest sense of the word so annoying that it can really become a problem, we already had that. However, this is not reproducible in the sense because it is up to this contact person. It can be gone very quickly."*
(LQ9)	*"And we would also like to see this sense of responsibility among our employees. That means they should take care of themselves a bit. You will of course get an introduction. They also get the annual refresher training. However, it is the case that we actually put great importance to the fact that they really look after themselves and also look after their colleagues. Not that we give up responsibility as such, but we want them to stay mentally fit. In all matters."*

(LQ10)	*"So, I think that might be the key challenge - there are no problems - with micro companies. What is probably often the case is that people say, yes, here, I am so close to everyone or I claim to be so close that I would rather take individual countermeasures than having a general rule, a measure, whatever I set up."*
(LQ11)	*"On the other hand, I can also imagine it, yes, but I think it's also a bit a question of size. So if three of you are sitting there, it's different from when you're growing a little bit into the size of a company around ten or so and maybe can't really look at everyone anymore and perhaps can't go there anymore, that you say you're going to be more systematic and regulated, analysing certain things in cycles and dealing with them, yes?"*
(LQ12)	*"The more you get, the bigger in theory – I don't want to call it necessity now - but the more sense it might make, for example in the area of working hours, to really impose structures, processes and measures that then / But (sighs) I can already see that on vacation and with a few other things, which people always run after, that they also do it and mhm, technical possibility yes, but then at every event it would have to be added manually will."*
(LQ13)	*"These events are one of the few things where there is clearly a work peak in terms of strain and so on and we have, well, not tons of them, but many. In this respect, it offered itself to take a measure or an automatism there. For all other things, what we have to work is so individual and also the challenges that can arise there are so individual that I don't see where certain risks could be identified or risk groups for which a measure could be deposited directly up front."*
(LQ14)	*"As a rule - that's why I was so surprised - that small businesses are exempt from almost all of these requirements. Luckily. So that's not necessarily related to occupational health and safety or risk assessment, but the administrative requirements with regard to everything are already quite extensive in Germany and that's another thing again to the steep learning curve, where also as a young*

	entrepreneur you don't necessarily have to look for more obligations that somehow entail an administrative burden."
(LQ15)	*"You said that it must theoretically also be documented and so on. At some point you document yourself to death and it's just that / so the added value is somehow not really there anymore."*
(LQ16)	*"(...) That we just go and say so once a week we run around and ask "how are you?". Or we have a questionnaire or more precisely, a questionnaire scheme, similar to an audit, like a quality audit. That we can just hand it over and go through it informally with employees, without it being an official audit. Rather, like an informal conversation, where we clarify everything is still right. That would be the wish. "*
(LQ17)	*"Because I believe that many smaller companies don't manage all these regulations, which one has anyway, with personal protective equipment and a face mask and all the junk, I say / junk, not to be seen negatively at this point. They just don't manage to do that because they simply don't have the power or the time or the capacity to do it. And that's why I think that a voluntary offer to the companies that relieves the companies on the spot and thus offers added value."*
(LQ18)	*"So, we also get the documents from the Profession Association, which regularly end up in our trash because nobody has time to read them. Not because it's not interesting, but we have enough work to do to keep the company afloat."*
(LQ19)	*"As I said, we are a production company. We produce, we manufacture parts. So that's the focus. Occupational safety, health and so on, yes, is one of them. But is not in direct focus. So not in the focus point. It is always up to date when something goes wrong in quotation marks. So when there is an accident at work or when several people are out and at the same time is still on vacation or something like that."*

(LQ20)	*"And if you are not there yourself - which is okay, I don't have to be there as a manager, because otherwise they may not speak openly – that I could then also direct the matter in the right direction, or trying to steer to the right direction - in my opinion."*
(LQ21)	*"So, risk assessment is actually the - heretical I would say - invention of an official who has no instinct of what he shouldn't do. So, risk assessment is just that someone assesses how great the risk is in my opinion for a certain activity or thing, with possible consequences."*
(LQ22)	*"If you are one of those, I say - the doer who somehow builds the company the way you think it is right, so of course you always want to be convinced of things and if I suggest it to someone, I would have to start to deal with it myself, yes - what areas are there at all? So then maybe I would realize, okay, there is a lot that I don't have on the agenda, and in the moment when I start to realise it, I would have the necessity to understand it and then maybe come up with the idea to implement it regularly, right? So, information, education."*
(LQ23)	*"Well, the law is one thing, but I think, sensitization is a much more interesting story than any stupid law. I have to be convinced that it will help me, that it will take me further, then I will do it and that can only be done through sensitization."*
(LQ24)	*"But I simply claim: In the trade sector, yes, if I were to go there with any campaign or I don't know what, approach companies, that they would smile and say: "Yes, well, that happens in other companies. But not to us."".*
(LQ25)	*"I think this overload depression or burn-out, or whatever you want to call it, is a thing that is elusive, especially for someone who hasn't had it. It's like trying to explain a violin to a pigeon. Or the colour red to a blind person who has never seen it before. You can only ever make comparisons. Like a tomato is red, but if he hasn't seen it, how should we do it? And if someone has never had this type of psychological stress and does not know it, then they cannot understand it. And then it's hard to explain. Even if I try to explain it with these pictures, you will*

	only ever look into questioning eyes and it can't be that bad. It is only bad when you have it yourself. Or have the experience with it."
(LQ26)	*"As a comparison, the law does not help that someone does not drive too fast, but rather the insight that fifty is appropriate for the city, soft fifty. That helps a lot more, so an understanding of what is it for, where does it make sense. And not, that's the law now, so you have to stick to it and you have to do it."*
(LQ27)	*"There are, I don't know, two or three simple questions, that probably sounds like - two or three simple questions won't work, but look – watch out for these areas in the company and if you see signs, we are happy to come and advise you."*
(LQ28)	*"I don't think all these questionnaires make sense, especially if I have to fill them out mechanically. I still think that's a case-by-case view. And from my point of view, these questionnaires are a reminder that I subsequently asked the best possible question. But this stoic ticking, also the traffic light system that is around - it's a difficult topic. However, I also noticed that these questionnaires are very focused on the trade sector - from that point of view. In addition, I think you just have to differentiate it. So, we have redesigned many, many things ourselves, also with regard to forms. Simply because you have to see it profession related."*
(LQ29)	*"And then again, there are so many laws and there are so many things, if you don't watch out that they will be implemented and that ultimately it will also be punished if it's not done, what use are they? (...) Again, if there's no plaintiff, there's no judge. And that's why - the laws exist and who implements them?"*
